# Computer-aided diagnosis of lung nodule using gradient tree boosting and Bayesian optimization

**DOI:** 10.1371/journal.pone.0195875

**Published:** 2018-04-19

**Authors:** Mizuho Nishio, Mitsuo Nishizawa, Osamu Sugiyama, Ryosuke Kojima, Masahiro Yakami, Tomohiro Kuroda, Kaori Togashi

**Affiliations:** 1 Department of Diagnostic Imaging and Nuclear Medicine, Kyoto University Graduate School of Medicine, Shogoin, Sakyo-ku, Kyoto, Kyoto, Japan; 2 Preemptive Medicine and Lifestyle Disease Research Center, Kyoto University Hospital, Shogoin, Sakyo-ku, Kyoto, Kyoto, Japan; 3 Department of Radiology, Osaka Medical College, Takatsuki, Osaka, Japan; 4 Department of Biomedical Data Intelligence, Kyoto University Graduate School of Medicine, Sakyo-ku, Kyoto, Kyoto, Japan; 5 Division of Medical Information Technology and Administrative Plannnig, Kyoto University Hospital, Shogoin, Sakyo-ku, Kyoto, Kyoto, Japan; Janssen Research and Development, UNITED STATES

## Abstract

We aimed to evaluate a computer-aided diagnosis (CADx) system for lung nodule classification focussing on (i) usefulness of the conventional CADx system (hand-crafted imaging feature + machine learning algorithm), (ii) comparison between support vector machine (SVM) and gradient tree boosting (XGBoost) as machine learning algorithms, and (iii) effectiveness of parameter optimization using Bayesian optimization and random search. Data on 99 lung nodules (62 lung cancers and 37 benign lung nodules) were included from public databases of CT images. A variant of the local binary pattern was used for calculating a feature vector. SVM or XGBoost was trained using the feature vector and its corresponding label. Tree Parzen Estimator (TPE) was used as Bayesian optimization for parameters of SVM and XGBoost. Random search was done for comparison with TPE. Leave-one-out cross-validation was used for optimizing and evaluating the performance of our CADx system. Performance was evaluated using area under the curve (AUC) of receiver operating characteristic analysis. AUC was calculated 10 times, and its average was obtained. The best averaged AUC of SVM and XGBoost was 0.850 and 0.896, respectively; both were obtained using TPE. XGBoost was generally superior to SVM. Optimal parameters for achieving high AUC were obtained with fewer numbers of trials when using TPE, compared with random search. Bayesian optimization of SVM and XGBoost parameters was more efficient than random search. Based on observer study, AUC values of two board-certified radiologists were 0.898 and 0.822. The results show that diagnostic accuracy of our CADx system was comparable to that of radiologists with respect to classifying lung nodules.

## Introduction

Lung cancer is the leading cause of cancer deaths in the United States [1] because it is frequently diagnosed at an advanced stage, and this prevents effective treatment. Results from the National Lung Screening Trial (NLST) show that compared with chest X-ray screening, lung cancer screening with low-dose CT significantly reduced lung cancer mortality among heavy smokers by detecting lung cancers at an early stage [2,3]. However, false positives in low-dose CT screening can be problematic and can result in unnecessary follow-up CT, positron emission tomography, or invasive procedures. In NLST, 96.4% of the positive results in the low-dose CT group were false positives [2,3].

Computer-aided diagnosis (CAD) has the potential of optimizing radiologists’ workloads. CAD can assist radiologists in detection (CADe) and differentiation (CADx) of lung nodules [4–23]. For example, CADx is useful for assisting radiologists in differentiating between benign and malignant lung nodules [6], and it is expected that CADx is useful for reducing false positives in lung cancer screening with low-dose CT.

Gradient tree boosting is superior to off-the-shelf classifiers such as random forest or support vector machine (SVM) [24,25]. Because performance of CADx is affected by machine learning algorithms, gradient tree boosting may improve the performance of CADx. However, to the best of our knowledge, no other study has investigated the usefulness of gradient tree boosting in CADx of lung nodules. In our study, we used XGBoost as an implementation of gradient tree boosting [25] and applied it to CADx system of lung nodules.

It is necessary to optimize parameters of machine learning algorithms to ensure good performance. Grid search has been frequently used for this purpose [26]. However, when the number of parameters is increased, grid search is not feasible because of its computational cost. As an alternative to grid search, random search and Bayesian optimization were used for parameter optimization [27,28]. Because XGBoost has many parameters, random search and Bayesian optimization are suitable for parameter optimization.

The purpose of the current study was to develop and evaluate the CADx system, focusing on (i) usefulness of the conventional CADx system (hand-crafted imaging feature + machine learning algorithm), (ii) comparison between SVM and XGBoost as machine learning algorithms, and (iii) effectiveness of parameter optimization using random search and Bayesian optimization. Herein, a variant of the local binary pattern (LBP) [11,18,29–31] was used as the hand-crafted imaging feature for calculating a feature vector that was fed into the machine learning algorithms.

## Methods

This study used anonymized data from a public database. Regulations of Japan did not require institutional review board approval for use of a public database.

### CT images

Our CADx system was tested using chest CT images obtained from The Cancer Imaging Archive (TCIA). TCIA is an open database of medical images, mainly consisting of CT, MRI, and nuclear medicine images that are stored as anonymized DICOM data. We used two sets of chest CT images from TCIA; one set from the LUNGx Challenge and one from the NSCLC Radiogenomics [20,21,32–35]. The LUNGx Challenge provided 60 test sets of chest CT images with 10 calibration sets. The 60 test sets included 73 lung nodules; a list of these nodules is available on the LUNGx Challenge website [34]. Among the 73 nodules from the LUNGx Challenge, 36 were lung cancers and 37 were benign. In NSCLC Radiogenomics, each of 26 sets of chest CT images included lung cancer. By combining data from LUNGx Challenge with those from NSCLC Radiogenomics, a total of 99 lung nodules (62 lung cancers and 37 benign nodules) were used for the development and evaluation of our CADx system.

### Image preprocessing

First, CT images were loaded, and their voxel sizes were converted to 1 × 1 × 1 mm. Next, the center was determined for each of the 99 nodules. Coordinates of the center of the lung nodules were provided via spreadsheet in the LUNGx Challenge and utilized here. Conversely, no such information was available for NSCLC Radiogenomics. Therefore, the center of the lung nodule was visually validated by two board-certified radiologists (M.N. and M.N.). A 64 × 64 × 64 3D bounding box was set for each nodule, and CT images inside the bounding box were cropped. The cropped 3D CT images were analyzed as the input to our CADx system. Areas of the CT images outside the bounding box were not assessed. As shown, manual segmentation of lung nodule was not needed in this preprocessing (only the center of lung nodule was necessary).

### Calculation of a feature vector

The local binary pattern on three orthogonal planes (LBP-TOP) was used for calculating a feature vector [11,18,29,30,31]. Naïve implementation of 2D LBP was represented as follows:

$$LBP(x,R,P)=\sum_{i=0}^{P-1}2^{i}\times s(d_{i})$$

$$d_{i}=I\left(n\left(x,R,i\right)\right)-I\left(x\right),$$ (I)

where *x* is the center pixel where LBP is calculated; *P* is the number of samples; *n*(*x*, *R*, *i*) is the *i*^th^ neighbor pixel around the center pixel *x* and the distance between the center pixel *x* and the neighbor pixel is *R*; *I*(*u*) is the CT density of pixel *u* and *s*(*v*) is an indicator function, where *s*(*v*) is 1 if *v ≥* 0 and 0 otherwise. We used a uniform pattern and rotation invariant type instead of naïve implementation as naïve implementation cannot handle large *P* values because they make feature vectors too long. Both uniform pattern and rotation invariant type can enhance the robustness of LBP as a feature vector. To utilize LBP in 3D images, LBP-TOP was used in this study. In LBP-TOP, 2D LBP was calculated on the XY, XZ and YZ planes and the texture information on other 3D planes was ignored. Then, the results of 2D LBP on the XY, XZ and YZ planes were converted into 1D histograms, which were concatenated. In this method, rotation invariance of LBP was retained only in the rotation of XY, XZ and YZ planes. To use LBP-TOP as feature vectors of CADx of lung nodules, 3D cropped CT images were evaluated with uniform pattern and rotation invariant type of LBP-TOP, and 1D feature vectors were calculated.

### Machine learning algorithm

Our CADx system was built using SVM or XGBoost [24,25]. Implementations of SVM and XGBoost were freely available. SVM or XGBoost were trained using the feature vector obtained by LBP-TOP and its corresponding label. SVM is a widely used machine learning algorithm, and we used SVM with kernel trick (radial basis function) in this study. XGBoost builds an efficient classifier using gradient tree boosting. Gradient tree boosting is invariant to scaling of a feature vector, and it can determine higher-order interaction between a feature vector. The usefulness of XGBoost has been validated in a number of machine learning and data mining challenges (Please refer to the Introduction section of [25]). Gradient tree boosting is trained in an additive manner. At each time step *t*, it grows a tree to minimize the residual of the current model. Formally, the objective function of XGBoost can be described as follows:

$$L^{t}=\sum_{i=1}^{n}l(y_{i},y_{i}^{t-1}+f_{t}(x_{i}))+\Omega (f_{t}),$$ (II)

where *x*_*i*_ and *y*_*i*_ are the feature vector and its label at the *i*^th^ instance, *n* is the number of training data, $y_{i}^{t-1}$ is the prediction of the *i*^*th*^ instance at the *t − 1*^*th*^ iteration, *f*_*t*_ is a new tree that classifies the *i*^th^ instance using *x*_*i*_, *l* denotes a loss function that measures the difference between the label and the prediction at the last step plus the new tree output, and *Ω* is the regularization term that penalizes the complexity of the new tree.

### Parameters

The following parameter space was used for parameter optimization.

- For SVM, *C* and **γ** were used for controlling SVM with a radial basis function kernel [26]; C is a parameter for balancing classification error and regularization, and **γ** is a free parameter for bandwidth of radial basis function kernel. The range of *C* and ***γ*** were as follows: *C*, 1.0 × 10^−5^–1.0 × 10^5^ and ***γ*,** 1.0 × 10^−5^–1.0 × 10^5^.
- For XGBoost, parameters and their range were as follows: eta, 0.2–0.6; max_depth, 1–13; min_child_weight, 1–10; gamma, 0–1. The concise explanation of XGBoost parameters are as follows: eta for step size shrinkage used in updating a tree [25], max_depth for maximum depth of a tree, min_child_weight for minimum sum of instance weight needed in a child (If the tree partition step results in a leaf node with the sum of instance weight less than min_child_weight, the process of tree building will stop further partitioning), gamma for minimum loss reduction required to make a further partition on a leaf node of the tree. The detail of these parameters can be available elsewhere [36].
- LBP-TOP has two parameters (*R* and *P*). The values of *R* and *P* were as follows: *R* = 7, 8 and *P* = 40, 48.

### Parameter optimization

The parameter space was defined in the previous subsection. Here we denoted the parameters as ***θ***. When using machine learning algorithm *A* (SVM or XGBoost) and the parameter ***θ***, we trained *A* using training data and validated its performance using validation data. We used *L*(*A*,***θ***,*D_train_*,*D_valid_*) to denote the validation loss that *A* achieved on validation data *D*_*valid*_ when *A* was trained on ***θ*** and *D*_*train*_. The parameter optimization problem under *K*-fold cross-validation was then to minimize the black box function:

$$f\left(\theta \right)=\frac{1}{K}\sum_{i=1}^{K}L\left(A,\theta ,D_{train}^{i},D_{valid}^{i}\right),$$ (III)

where $D_{train}^{i}$ and $D_{valid}^{i}$ were training data and validation data of the *i-th* fold of *K*-fold cross-validation, respectively. Bayesian optimization was used for optimizing this black box function *f*(***θ***) and for searching for the optimal parameter ***θ***. Tree Parzen Estimator (TPE) was utilized for solving this problem [27]. Random search was used to compare the performance of TPE. Number of trials for TPE or random search was as follows: 10, 100, 200, and 1000.

### Software and outline of CADx

**Fig 1:**
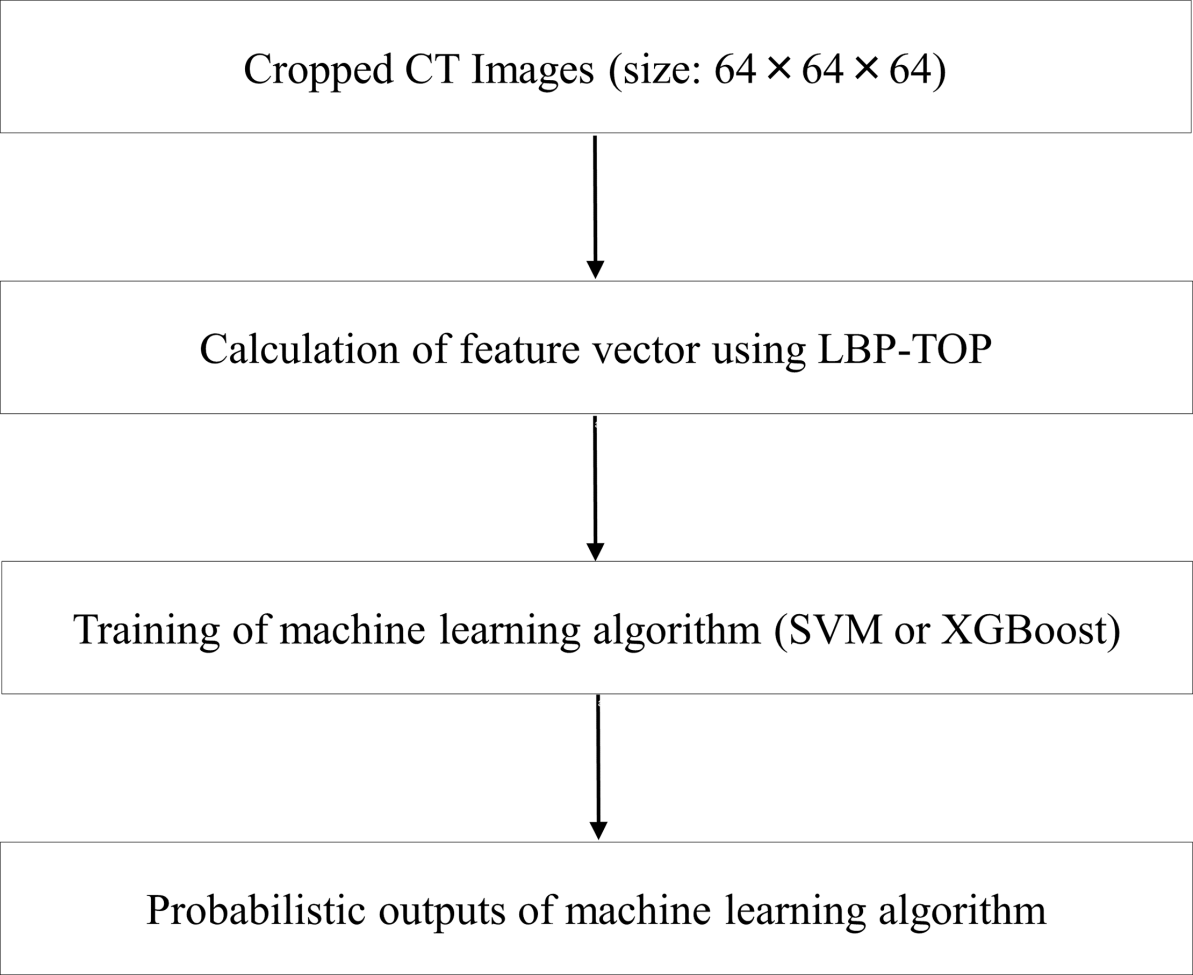
Outline of our CADx system. Abbreviations: CADx, computer-aided diagnosis; LBP-TOP, local binary pattern on three orthogonal planes; SVM, support vector machine.

Code of our CADx system and binary data of lung nodules are available as S1 File of Supporting information. For our CADx system, python (version 2.7, https://www.python.org/), and the following packages were used: scikit-image (version 0.18.1, http://scikit-image.org/), sciki-learn (version 0.18.1, http://scikit-learn.org/), xgboost (version 0.6, http://xgboost.readthedocs.io/en/latest/), and hyperopt (version 0.1, http://hyperopt.github.io/hyperopt/). An outline of our CADx system is shown in Fig 1.

### Observer study

Two board-certified radiologists (M.N. and M.Y.) were included in observer study for assessing the 99 lung nodules. They evaluated CT images of lung nodules with lung window condition (window width = 1500 HU and window level = −600 HU), and could change the window condition if necessary. They rated their suspicion of malignancy with 10-point scale (1 = definitely benign lung nodule; 10 = definitely lung cancer).

### Statistical analysis

Leave-one-out cross-validation was used for optimizing and evaluating the performance of our CADx system. Validation loss under leave-one-out cross-validation was used for parameter optimization. After parameter optimization, probabilistic outputs of our CADx system with optimal parameters were analyzed using accuracy and area under the curve (AUC) of receiver operating characteristic (ROC) analysis. Classification results of our CADx system were output as probabilities of lung cancer to calculate AUC. For each number of trial, AUC and accuracy were calculated 10 times, and their averages were obtained. For observer study, AUC and accuracy of the two board-certified radiologists were also calculated for comparison between our CADx system and radiologists.

## Results

**Fig 2:**
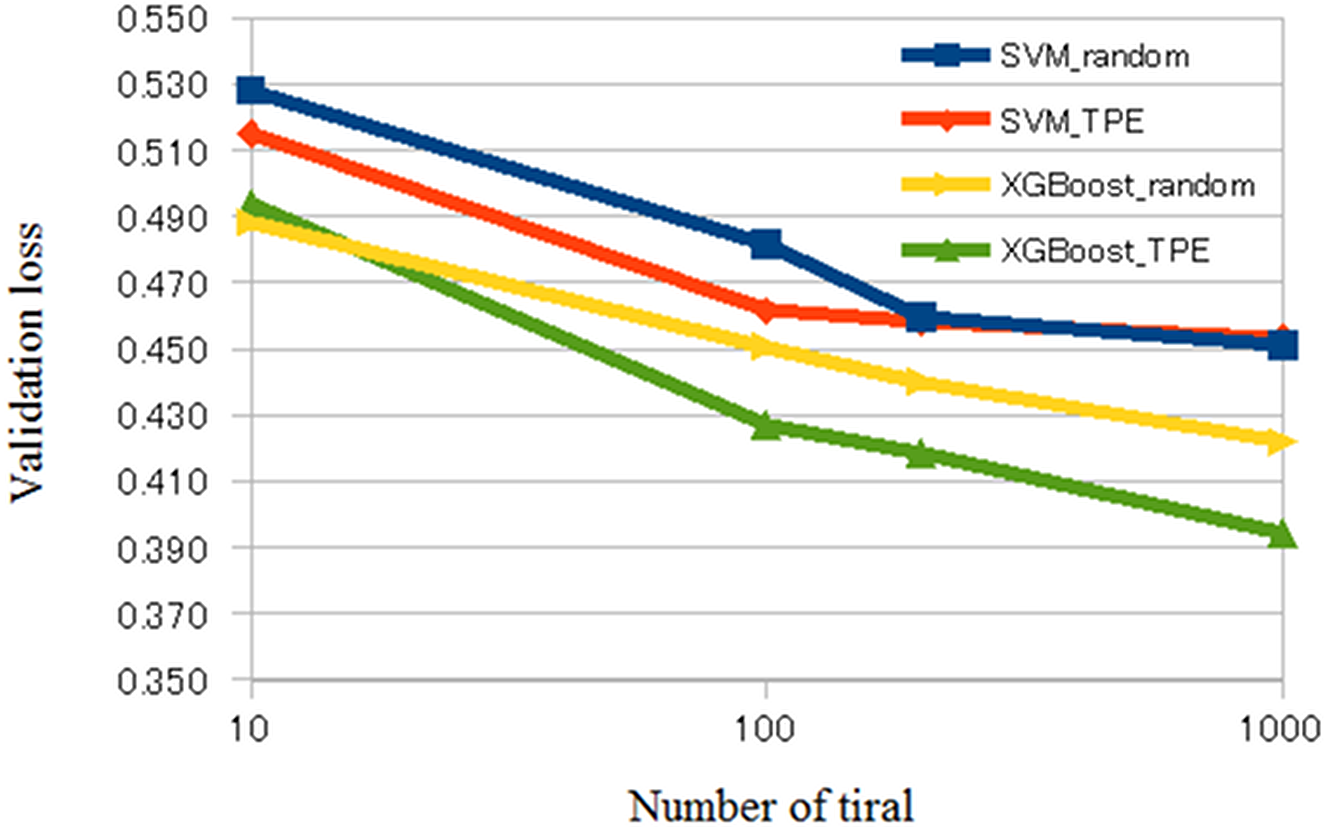
Validation loss of CADx. Abbreviations: CADx, computer-aided diagnosis; SVM, support vector machine.

**Fig 3:**
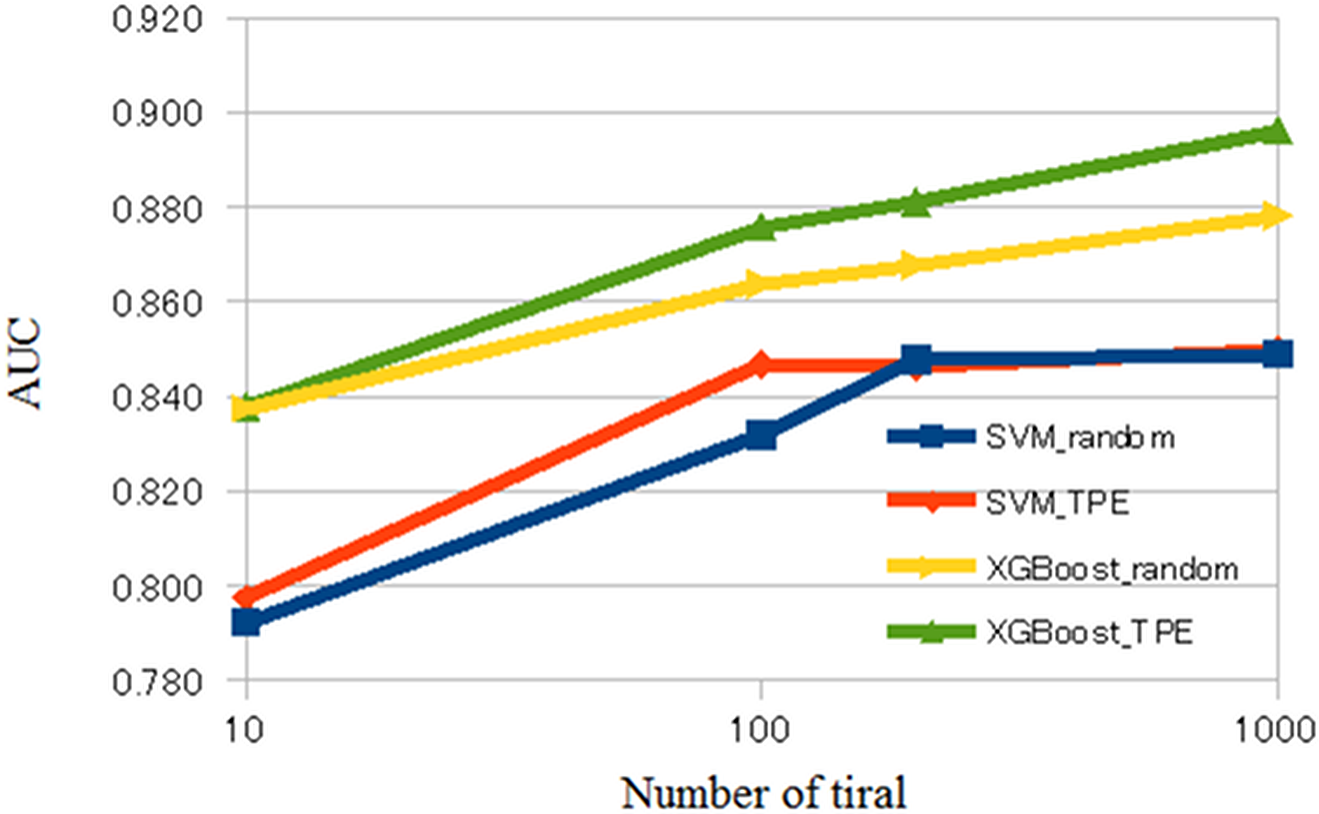
AUC of CADx. Abbreviations: CADx, computer-aided diagnosis; SVM, support vector machine; AUC, area under the curve.

**Fig 4:**
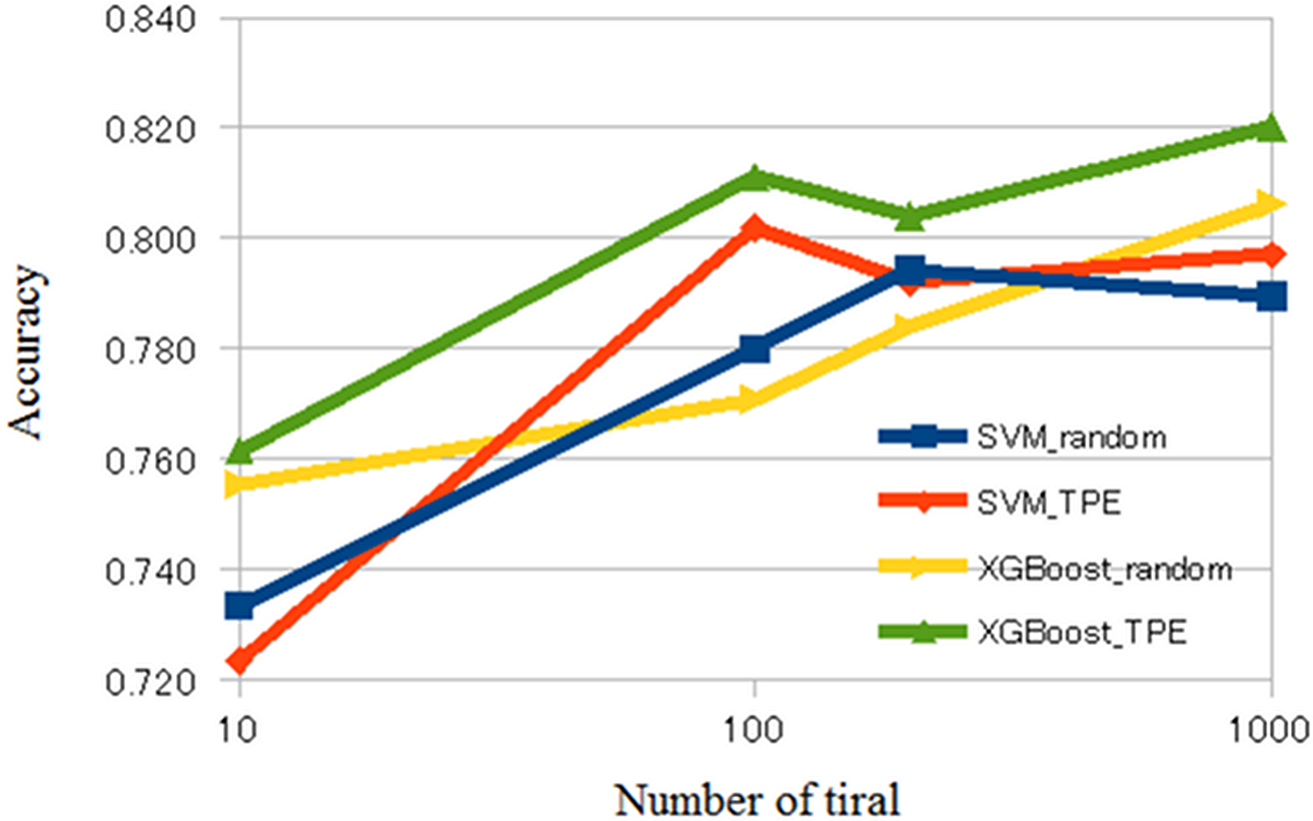
Accuracy of CADx. Abbreviations: CADx, computer-aided diagnosis; SVM, support vector machine.

The averaged validation loss, AUC, and accuracy of our CADx system are shown in Tables 1 and 2 and Figs 2–4. S1 Table of Supporting information shows raw results of validation loss, AUC, and accuracy of our CADx system for each setting. Tables 1 and 2 show the averages of the raw results listed in S1 Table of Supporting information. Comparing the results depicted in Tables 1 and 2, XGBoost was generally superior to SVM. According to Table 1, the best averaged AUC of SVM was 0.850 when using TPE and number of trials = 1000. Table 2 shows that the best averaged AUC of XGBoost was 0.896 when using TPE and number of trials = 1000. According to S1 Table of Supporting information, the best AUC and accuracy of SVM was 0.855 and 0.834, respectively, and the best AUC and accuracy of XGBoost was 0.903 and 0.859, respectively. In XGBoost, the averaged AUC of TPE was better than that of random search when the number of trials was 100, 200, or 1000. In SVM, the averaged AUC of TPE was better than that of the random search when the number of trials was 100. However, when the number of trials was 10, the difference of the averaged AUC was minimal between random search and TPE in SVM and XGBoost. In addition, in SVM, the difference of averaged AUC was minimal between random search and TPE when the number of trials was 200 or 1000.

**Table 1:** Results of CADx when using SVM and parameter optimization.

Algorithm	Number of trial	Validation loss	AUC	Accuracy
Random	10	0.528	0.792	0.734
Random	100	0.481	0.832	0.780
Random	200	0.460	0.848	0.794
Random	1000	0.451	0.849	0.789
TPE	10	0.515	0.797	0.724
TPE	100	0.461	0.847	0.802
TPE	200	0.458	0.846	0.792
TPE	1000	0.453	0.850	0.797

Abbreviation: computer-aided diagnosis, CADx; support vector machine, SVM; Tree Parzen Estimator, TPE; area under the curve, AUC.

**Table 2:** Results of CADx when using XGBoost and parameter optimization.

Algorithm	Number of trial	Validation loss	AUC	Accuracy
Random	10	0.488	0.838	0.756
Random	100	0.451	0.864	0.771
Random	200	0.440	0.868	0.784
Random	1000	0.422	0.878	0.806
TPE	10	0.494	0.838	0.762
TPE	100	0.427	0.876	0.811
TPE	200	0.419	0.881	0.804
TPE	1000	0.394	0.896	0.820

Abbreviation: computer-aided diagnosis, CADx; support vector machine, SVM; Tree Parzen Estimator, TPE; area under the curve, AUC.

**Fig 5:**
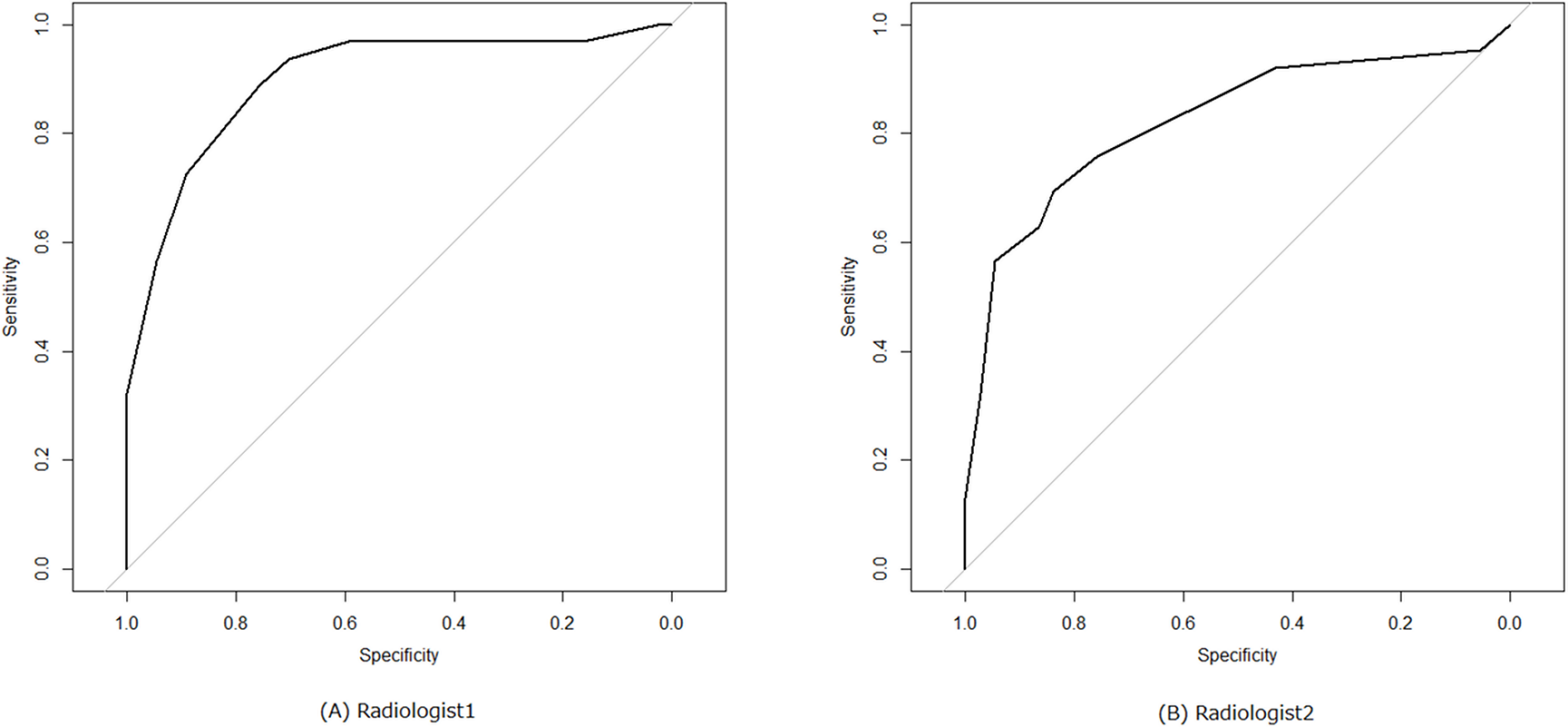
ROC curves of two radiologists. Note: (A) radiologist1 and (B) radiologist2. Abbreviations: ROC, receiver operating characteristic.

From the results of observer study, AUC and accuracy of the two board-certified radiologists were as follows: radiologist1, 0.898 and 0.838; radiologist2, 0.822 and 0.717. Fig 5 shows the corresponding ROC curves of the two radiologists.

## Discussion

In this study, we used two different sets of CT images for evaluating our CADx system; one set from the LUNGx Challenge and the other from the NSCLC Radiogenomics. Using XGBoost and TPE, the best averaged AUC under leave-one-out cross-validation was 0.896 (the best AUC under leave-one-out cross-validation was 0.903). AUC values of the two board-certified radiologists were 0.898 and 0.822. These results of our CADx system show the following three main points; (i) the diagnostic accuracy of our conventional CADx system (hand-crafted imaging feature + machine learning algorithm) might be comparable to that of the radiologists; (ii) XGBoost was better than SVM; and (iii) parameter optimization with TPE was better than that with random search.

From the comparison between our CADx system and radiolgists, we speculated that the diagnostic accuracy of our CADx system was comparable to that of the radiologists with respect to classifying lung nodules; AUC values of both our CADx system and radiologists were nearly 0.9.

A few previous studies have utilized XGBoost for developing a clinical model. One study used XGBoost for classifying symptom severity based on text information in the form of psychiatrist notes [37]. Another study showed the usefulness of XGBoost for differentiation between subjects with epilepsy and healthy subjects using patients’ cerebral activity assessed by functional MRI [38]. In conjunction with the results of these studies, we found that XGBoost was useful for developing an efficient and reliable clinical model. Although SVM was widely used as a machine learning algorithm in CADx, AUC of CADx using XGBoost was better than that using SVM in our study. A prime reason for the superiority of XGBoost to SVM is invariant to scaling of a feature vector. As well-known kernels for SVM, such as radial basis function and linear kernels, are scale dependent, the output value of SVM is affected by scaling of a feature vector. In addition, because results of LBP-TOP can be viewed as a type of categorical data, it is speculated that tree-based classifiers, such as XGBoost, are more suitable for feature vectors obtained by LBP-TOP than SVM.

Previous studies have shown that Bayesian optimization was useful in several domains of clinical application [39–42]. The results of the current study are compatible with those of the previous studies. Figs 2–4 show that, in general, TPE is better than random search for optimizing parameters in SVM and XGBoost. However, when the number of trials was 10, the difference in performance between TPE and random search was minimal. This result suggests that the small number of trials (10) hindered parameter optimization of SVM and XGBoost. When the number of trials was 200 or 1000 in SVM, the difference in performance between random search and TPE was also minimal. Because parameter space of SVM was narrower than that of XGBoost in the current study, we surmised that both random search and TPE could almost fully optimize parameters and the difference in performance may be minimal.

We used the conventional CADx system because the number of lung nodule was less than 100. Although results of recent studies suggest that deep learning is superior to conventional machine learning algorithms, deep learning requires a large number of training data [43]. Therefore, we focused on the conventional CADx system in the current study. Generally speaking, it is more difficult to collect training data for medical image analysis than for other fields of image analysis. When the number of training data is limited, our methodology may be more useful than deep learning.

Because we used the established software (LBP, XGBoost, and TPE), our CADx system was technically simple. However, our results show that diagnostic accuracy of our system might be comparable to that of the radiologists. The previous studies required nodule segmentation, calculation of many types of image features, or radiological findings for successful differentiation of lung nodules [8, 17]. The simplicity was the main advantage of our CADx system.

The previous study shows that AUC value of CADx system was more than 0.8 by using the 73 lung nodules of LUNGx Challenge and SVM with a linear kernel [11]. Because of differences in CT images, quality of labels, and kernel type of SVM, it is difficult to precisely compare the diagnostic accuracy of the CADx system between the current study and the previous study. However, the diagnostic accuracy of our CADx system using SVM might be comparable to that of the previous study.

There were several limitations to our study. First, the number of lung nodules was relatively small. Hence, our CADx system might overfit the dataset of the current study. We speculated that the possibility of overfitting was not so high because this dataset consisted of two different sets of CT images and the conditions and parameters of the images were variable (i.e. variability in use of contrast material and thickness of CT images). However, this speculation might be optimistic as we cannot deny a possibility that our CADx system overfitted the dataset of the current study. Future studies should be conducted using a large number of lung nodules to prevent overfitting and evaluate the generalizability of our CADx system. Second, this study focused on the investigation of technical usefulness of XGBoost and Bayesian optimization from the viewpoint of CADx of lung nodules, and we ignored the clinical usefulness of our CADx system. Because the results of our study showed that the diagnostic ability of our CADx system may be comparable to that of radiologists, we expect that our CADx system will be useful for classifying lung nodules in a practical clinical setting. Third, the parameter space was relatively limited in this study. The parameters of our study were divided into two types: the parameter of machine learning algorithms (i.e. *C* for SVM and eta for XGBoost) and the parameter of feature vectors (*R* and *P* of LBP). Because the results of parameter optimization were not stable when the parameter space of feature vectors was wide, we restricted the parameter space of feature vectors in our study. Last, we did not compare our CADx system with CADx using deep learning. We plan to develop a CADx system with deep learning and will use TPE for parameter optimization of deep learning in a future study.

In conclusion, XGBoost was better than SVM for classifying lung nodules. For optimizing parameters of both SVM and XGBoost, Bayesian optimization was more efficient than random search. Although our results were preliminary, the diagnostic accuracy of our CADx system may be comparable to that of radiologists for classifying lung nodules.

## Supporting information

S1 Table Raw results of parameter optimization. S1 Table shows the raw results of validation loss, AUC, accuracy of our CADx system for each setting. Tables 1 and 2 show the averages of the raw results.
(DOCX)

S1 File Code of our CADx system and binary data of lung nodules. S1 File includes Python script of our CADx system and binary data of lung nodules stored as NPY.
(ZIP)
