# Supplementary material for: Computer-aided diagnosis of lung nodule using gradient tree boosting and Bayesian optimization
Source: PLoS One. 2018 Apr 19;13(4):e0195875. doi: 10.1371/journal.pone.0195875 (PMC5908232; doi:10.1371/journal.pone.0195875)
Supplement: S1 Table — shows the raw results of validation loss, AUC, accuracy of our CADx system for each setting. Tables 1 and 2 show the averages of the raw results. (DOCX) [file pone.0195875.s001.docx]

S1 Table

Raw results of parameter optimization.

Table S1 shows the raw results of validation loss, AUC, accuracy of our CADx system for each setting. Tables 1 and 2 show the averages of the raw results.

**SVM**

| Algorithm | Number of trial | Validation loss | AUC | Accuracy |
| --- | --- | --- | --- | --- |
| Random | 10 |  |  |  |
|  |  | 0.472852459 | 0.838905841 | 0.816161616 |
|  |  | 0.467337894 | 0.847689625 | 0.786868687 |
|  |  | 0.494684872 | 0.828814298 | 0.796969697 |
|  |  | 0.52000721 | 0.785483871 | 0.718181818 |
|  |  | 0.528832919 | 0.782279861 | 0.67979798 |
|  |  | 0.564000536 | 0.759873583 | 0.739393939 |
|  |  | 0.579100753 | 0.749498692 | 0.644444444 |
|  |  | 0.590203773 | 0.734982563 | 0.651515152 |
|  |  | 0.561277597 | 0.771403662 | 0.722222222 |
|  |  | 0.501580928 | 0.823931997 | 0.77979798 |
|  |  |  |  |  |
| Random | 100 |  |  |  |
|  |  | 0.453996306 | 0.84901918 | 0.782828283 |
|  |  | 0.486465281 | 0.824498692 | 0.758585859 |
|  |  | 0.463673017 | 0.85494769 | 0.782828283 |
|  |  | 0.50605269 | 0.810200523 | 0.766666667 |
|  |  | 0.466599725 | 0.841695728 | 0.818181818 |
|  |  | 0.491819683 | 0.827550131 | 0.786868687 |
|  |  | 0.478281981 | 0.83777245 | 0.772727273 |
|  |  | 0.500108052 | 0.816455972 | 0.758585859 |
|  |  | 0.499181226 | 0.812925022 | 0.75959596 |
|  |  | 0.468416559 | 0.842676548 | 0.813131313 |
|  |  |  |  |  |
| Random | 200 |  |  |  |
|  |  | 0.449465093 | 0.850196164 | 0.802020202 |
|  |  | 0.462081879 | 0.846599826 | 0.816161616 |
|  |  | 0.459725217 | 0.846752398 | 0.818181818 |
|  |  | 0.458060719 | 0.845727986 | 0.780808081 |
|  |  | 0.466087248 | 0.85065388 | 0.792929293 |
|  |  | 0.459916646 | 0.842720139 | 0.782828283 |
|  |  | 0.453127683 | 0.846272886 | 0.787878788 |
|  |  | 0.4727752 | 0.843221447 | 0.778787879 |
|  |  | 0.466469114 | 0.852462947 | 0.770707071 |
|  |  | 0.448841497 | 0.853160418 | 0.811111111 |
|  |  |  |  |  |
| Random | 1000 |  |  |  |
|  |  | 0.453286279 | 0.846534438 | 0.786868687 |
|  |  | 0.450903419 | 0.848997384 | 0.791919192 |
|  |  | 0.450341396 | 0.8494551 | 0.791919192 |
|  |  | 0.449242449 | 0.8505449 | 0.786868687 |
|  |  | 0.451700382 | 0.848256321 | 0.784848485 |
|  |  | 0.449170274 | 0.849585876 | 0.794949495 |
|  |  | 0.452971763 | 0.848953793 | 0.78989899 |
|  |  | 0.45200431 | 0.849585876 | 0.785858586 |
|  |  | 0.452164666 | 0.848125545 | 0.785858586 |
|  |  | 0.450675727 | 0.849476896 | 0.795959596 |
| TPE | 10 |  |  |  |
|  |  | 0.517580418 | 0.788600697 | 0.686868687 |
|  |  | 0.529115386 | 0.785876199 | 0.694949495 |
|  |  | 0.515797145 | 0.809568439 | 0.766666667 |
|  |  | 0.550014703 | 0.754163034 | 0.670707071 |
|  |  | 0.516836166 | 0.784045336 | 0.675757576 |
|  |  | 0.529077427 | 0.775915432 | 0.719191919 |
|  |  | 0.454394539 | 0.849629468 | 0.790909091 |
|  |  | 0.532519961 | 0.795880558 | 0.737373737 |
|  |  | 0.49796965 | 0.830122058 | 0.762626263 |
|  |  | 0.503909225 | 0.79978204 | 0.731313131 |
|  |  |  |  |  |
| TPE | 100 |  |  |  |
|  |  | 0.473905684 | 0.848910201 | 0.824242424 |
|  |  | 0.453385082 | 0.846730602 | 0.796969697 |
|  |  | 0.477545741 | 0.850741064 | 0.821212121 |
|  |  | 0.457103762 | 0.845836966 | 0.811111111 |
|  |  | 0.45151311 | 0.8505449 | 0.797979798 |
|  |  | 0.454227321 | 0.845989538 | 0.787878788 |
|  |  | 0.457181199 | 0.847210113 | 0.809090909 |
|  |  | 0.449047705 | 0.852244987 | 0.798989899 |
|  |  | 0.453109634 | 0.849171752 | 0.794949495 |
|  |  | 0.487489092 | 0.829555362 | 0.777777778 |
|  |  |  |  |  |
| TPE | 200 |  |  |  |
|  |  | 0.449103032 | 0.851198779 | 0.78989899 |
|  |  | 0.450863972 | 0.84989102 | 0.792929293 |
|  |  | 0.449983732 | 0.851438535 | 0.787878788 |
|  |  | 0.452206302 | 0.848038361 | 0.78989899 |
|  |  | 0.451227635 | 0.84923714 | 0.793939394 |
|  |  | 0.486096436 | 0.834459459 | 0.8 |
|  |  | 0.450834341 | 0.851002616 | 0.807070707 |
|  |  | 0.45350115 | 0.846512642 | 0.801010101 |
|  |  | 0.452603367 | 0.846948561 | 0.785858586 |
|  |  | 0.482127102 | 0.831560593 | 0.771717172 |
|  |  |  |  |  |
| TPE | 1000 |  |  |  |
|  |  | 0.448719475 | 0.852484743 | 0.814141414 |
|  |  | 0.451453543 | 0.849520488 | 0.793939394 |
|  |  | 0.451718977 | 0.848866609 | 0.793939394 |
|  |  | 0.478046461 | 0.849040976 | 0.834343434 |
|  |  | 0.451485383 | 0.849716652 | 0.782828283 |
|  |  | 0.448444877 | 0.851002616 | 0.788888889 |
|  |  | 0.450218994 | 0.848605057 | 0.783838384 |
|  |  | 0.449448807 | 0.848234525 | 0.784848485 |
|  |  | 0.451326652 | 0.850479512 | 0.787878788 |
|  |  | 0.449786465 | 0.851830863 | 0.806060606 |
|  |  |  |  |  |

**XGboost**

| Algorithm | Number of trial | Validation loss | AUC | Accuracy |
| --- | --- | --- | --- | --- |
| Random | 10 |  |  |  |
|  |  | 0.445145341 | 0.865736704 | 0.797979798 |
|  |  | 0.482081523 | 0.838709677 | 0.777777778 |
|  |  | 0.490143274 | 0.841325196 | 0.757575758 |
|  |  | 0.454091127 | 0.860505667 | 0.808080808 |
|  |  | 0.527766411 | 0.806887533 | 0.737373737 |
|  |  | 0.467754416 | 0.848735833 | 0.757575758 |
|  |  | 0.521778064 | 0.810810811 | 0.686868687 |
|  |  | 0.471237447 | 0.846556234 | 0.797979798 |
|  |  | 0.539348503 | 0.817349608 | 0.717171717 |
|  |  | 0.483087281 | 0.838709677 | 0.717171717 |
|  |  |  |  |  |
| Random | 100 |  |  |  |
|  |  | 0.446691798 | 0.869659983 | 0.767676768 |
|  |  | 0.436859639 | 0.869224063 | 0.787878788 |
|  |  | 0.473479152 | 0.850043592 | 0.717171717 |
|  |  | 0.458098385 | 0.850043592 | 0.777777778 |
|  |  | 0.446666752 | 0.857454228 | 0.757575758 |
|  |  | 0.44378296 | 0.868352223 | 0.777777778 |
|  |  | 0.458026174 | 0.857454228 | 0.757575758 |
|  |  | 0.445379934 | 0.872711421 | 0.757575758 |
|  |  | 0.452673391 | 0.869659983 | 0.797979798 |
|  |  | 0.444972962 | 0.870531822 | 0.808080808 |
|  |  |  |  |  |
| Random | 200 |  |  |  |
|  |  | 0.443760611 | 0.863121186 | 0.767676768 |
|  |  | 0.444146966 | 0.868788143 | 0.777777778 |
|  |  | 0.424888947 | 0.87532694 | 0.777777778 |
|  |  | 0.445441904 | 0.857018309 | 0.787878788 |
|  |  | 0.446177996 | 0.861813426 | 0.767676768 |
|  |  | 0.437196368 | 0.869224063 | 0.797979798 |
|  |  | 0.461010096 | 0.858326068 | 0.797979798 |
|  |  | 0.430482423 | 0.877506539 | 0.787878788 |
|  |  | 0.429184647 | 0.87401918 | 0.777777778 |
|  |  | 0.437877671 | 0.872275501 | 0.797979798 |
|  |  |  |  |  |
| Random | 1000 |  |  |  |
|  |  | 0.434565969 | 0.862685266 | 0.808080808 |
|  |  | 0.387608598 | 0.894507411 | 0.838383838 |
|  |  | 0.437005104 | 0.870531822 | 0.757575758 |
|  |  | 0.431226879 | 0.878814298 | 0.828282828 |
|  |  | 0.418224606 | 0.883609416 | 0.808080808 |
|  |  | 0.426340077 | 0.873583261 | 0.828282828 |
|  |  | 0.431543871 | 0.878378378 | 0.797979798 |
|  |  | 0.412123485 | 0.884481255 | 0.808080808 |
|  |  | 0.419350243 | 0.877506539 | 0.808080808 |
|  |  | 0.419302571 | 0.876198779 | 0.777777778 |
|  |  |  |  |  |
| TPE | 10 |  |  |  |
|  |  | 0.500940918 | 0.83391456 | 0.777777778 |
|  |  | 0.437500892 | 0.865736704 | 0.787878788 |
|  |  | 0.486661284 | 0.832170881 | 0.747474747 |
|  |  | 0.482096219 | 0.841761116 | 0.767676768 |
|  |  | 0.4862092 | 0.836094159 | 0.757575758 |
|  |  | 0.501471978 | 0.843504795 | 0.757575758 |
|  |  | 0.501419561 | 0.827375763 | 0.737373737 |
|  |  | 0.566848912 | 0.796425458 | 0.747474747 |
|  |  | 0.491636458 | 0.841325196 | 0.767676768 |
|  |  | 0.480854742 | 0.858326068 | 0.767676768 |
|  |  |  |  |  |
| TPE | 100 |  |  |  |
|  |  | 0.399637731 | 0.897994769 | 0.858585859 |
|  |  | 0.441848314 | 0.872275501 | 0.787878788 |
|  |  | 0.445585612 | 0.867044464 | 0.797979798 |
|  |  | 0.435037646 | 0.868352223 | 0.797979798 |
|  |  | 0.429655877 | 0.877942459 | 0.808080808 |
|  |  | 0.397312074 | 0.891455972 | 0.828282828 |
|  |  | 0.44651141 | 0.859633827 | 0.808080808 |
|  |  | 0.417638445 | 0.880122058 | 0.828282828 |
|  |  | 0.435326973 | 0.870967742 | 0.767676768 |
|  |  | 0.420929913 | 0.871403662 | 0.828282828 |
|  |  |  |  |  |
| TPE | 200 |  |  |  |
|  |  | 0.397735901 | 0.903225806 | 0.818181818 |
|  |  | 0.436144729 | 0.868788143 | 0.767676768 |
|  |  | 0.405300595 | 0.886660854 | 0.848484848 |
|  |  | 0.423472902 | 0.873583261 | 0.797979798 |
|  |  | 0.428297094 | 0.880557977 | 0.787878788 |
|  |  | 0.41926367 | 0.884045336 | 0.808080808 |
|  |  | 0.416707359 | 0.879250218 | 0.797979798 |
|  |  | 0.425973878 | 0.872275501 | 0.808080808 |
|  |  | 0.428851232 | 0.87401918 | 0.777777778 |
|  |  | 0.404465247 | 0.889276373 | 0.828282828 |
|  |  |  |  |  |
| TPE | 1000 |  |  |  |
|  |  | 0.402657802 | 0.89581517 | 0.777777778 |
|  |  | 0.378864325 | 0.900174368 | 0.858585859 |
|  |  | 0.379654844 | 0.901918047 | 0.858585859 |
|  |  | 0.404564335 | 0.894071491 | 0.777777778 |
|  |  | 0.380837913 | 0.900610288 | 0.858585859 |
|  |  | 0.420429095 | 0.881865737 | 0.808080808 |
|  |  | 0.383862273 | 0.897558849 | 0.848484848 |
|  |  | 0.403646672 | 0.894507411 | 0.777777778 |
|  |  | 0.384985044 | 0.900610288 | 0.858585859 |
|  |  | 0.404562696 | 0.894071491 | 0.777777778 |
|  |  |  |  |  |
